# Characterization of Large-Energy-Bandgap Methylammonium Lead Tribromide (MAPbBr_3_) Perovskite Solar Cells

**DOI:** 10.3390/nano13071152

**Published:** 2023-03-24

**Authors:** Mijoung Kim, Jungyup Yang

**Affiliations:** The Institute of Basic Science, Department of Physics, Kunsan National University, Gunsan 54150, Republic of Korea; kmj0602@kunsan.ac.kr

**Keywords:** perovskite solar cells, anti-solvent treatment, large energy bandgap, MAPbBr_3_

## Abstract

We have investigated the effects of the methylammonium bromide (MABr) content of the precursor solution on the properties of wide-bandgap methylammonium lead tribromide (MAPbBr_3_) perovskite solar cells (PSCs). In addition, the anti-solvent process for fabricating MAPbBr_3_ perovskite thin films was optimized. The MAPbBr_3_ precursor was prepared by dissolving MABr and lead bromide (PbBr_2_) in N,N-dimethylformamide and N,N-dimethyl sulfoxide. Chlorobenzene (CB) was used as the anti-solvent. We found that both the morphology of the MAPbBr_3_ layer and the PSCs performance are significantly affected by the MABr content in perovskite precursor solution and anti-solvent dripping time. The best-performing device was obtained when the molar ratio of MABr:PbBr_2_ was 1:1 and the CB drip time was 10 s. The best device exhibited a power conversion efficiency of 7.58%, short-circuit current density of 7.32 mA·cm^−2^, open-circuit voltage of 1.30 V, and fill factor of 79.87%.

## 1. Introduction

Due to ongoing global environmental problems, society is committed to developing clean and renewable energy sources. Renewable energy sources are considered a viable solution to improve the energy system and mitigate environmental problems. The major renewable energy sources include wind power, hydropower, solar energy, geothermal energy, thermoelectric power, energy harvesting using phase change materials, and bioenergy, among others [1,2,3,4,5,6,7,8]. Solar cells, in particular, have gained significant attention as a practical means of securing new and renewable energy, given their industrialization. Solar cells have low pollution levels, an infinite resource, and a semi-permanent lifespan, making them a promising energy source to solve future energy problems. Researchers are investigating various types of solar cells classified by material and structure to discover new photovoltaic materials capable of generating infinite sunlight. Examples include thin-film silicon solar cells, dye-sensitized solar cells (DSSC), quantum-dot solar cells, organic solar cells, compound semiconductor thin-film solar cells, and multi-junction solar cells [9]. However, multi-junction solar cells are used only in specific areas due to the high cost of device fabrication. DSSCs, quantum-dot solar cells, and organic solar cells have a great barrier to commercialization due to their low efficiency. To address the low-efficiency problem, thin-film photovoltaic cells were developed based on organic-inorganic halide perovskite (OIHP) absorbers. In addition, research has recently been conducted to improve their performance by extending the use of OIHP materials to a two-dimensional (2D) form. The 2D OIHP possess outstanding absorption properties, charge carrier transport, intense photoluminescence, and easy fabrication, making them the subject of enormous attention and widespread applications in high-performance optoelectronic devices [10].

Perovskite solar cells (PSCs) are the most promising light-absorbing materials among next-generation solar cell materials that will succeed silicon solar cells. Since OIHP was first used as a solar cell in 2009, OIHP PSCs have reached high efficiencies of over 25% from an initial efficiency of 3% [11]. The OIHP materials used for fabricating solar cells have many advantages, such as direct band gap properties, low exciton binding energy, long electron-hole diffusion length, narrow bandwidth, light-absorbing coefficient, and tunable bandgap properties [12,13,14,15,16,17]. Because of these characteristics, OIHP PSCs are not only applicable for light-harvesting devices but also have the potential for many other devices such as perovskite light-emitting diodes (PeLEDs), light sensors, highly-sensitive photodetectors, lasers, and transistors [18,19]. OIHP are low-cost materials and can be fabricated using a solution-based process constructed from all abundant materials and inexpensive sources [20,21]. The most widely studied OIHP materials are based on methylammonium lead tri-iodide (MAPbI_3_) [22,23]. However, methylammonium lead tri-bromide (MAPbBr_3_) has also gained significant attention due to its wide bandgap [24,25,26,27]. The deposition or coating of a uniform and dense MAPbBr_3_ OIHP thin film is challenging due to the unknown growth and microstructure relationship required to achieve high quality. Additionally, achieving a uniform, dense, and flat coating of the MAPbBr_3_ thin film is difficult, so many researchers select two-step spin-coating, single crystal growth method, sequential deposition, and one-step spin coating using anti-solvent engineering, taking cues from the high-quality MAPbI_3_ thin film formation process [28,29,30,31,32].

For transparent photovoltaic devices, it is possible to fabricate them by tuning the band gap and using different solar cell architectures, such as creating a thin absorber layer with a large energy bandgap. Transparent photovoltaic devices can serve as windows in buildings, providing both shading and green electricity for the building. The wide bandgap of MAPbBr_3_ allows light partially in the visible region to pass through the perovskite film. In addition to its use as a single junction, MAPbBr_3_ can also be utilized as the top cell in double or triple-junction solar cells because it acts as a window, transmitting the long wavelength of ~534 nm and above spectrum to the bottom cell for photogeneration [33].

In this study, we utilized the most common and straightforward method for fabricating perovskite thin film in the laboratory, namely one step spin coating on a planar substrate. We fabricated MAPbBr_3_ solar cells using an n-i-p normal planar structure consisting of fluorine-doped tin oxide (FTO)/TiO_2_/MAPbBr_3_/Spiro-OMeTAD/Ag. TiO_2_ and Spiro-OMeTAD were the electron transport layer (ETL) and hole transport layer (HTL), respectively. We investigated the properties of the MAPbBr_3_ thin films and devices formed from precursor solutions with various methylammonium-bromide (MABr) contents. Additionally, we examined the quality of MAPbBr_3_ thin films formed through anti-solvent (chlorobenzene or CB) engineering [34] using an optimized MABr:PbBr_2_ molar ratio. Our results showed that altering the molar ratios of MABr:PbBr_2_ and the anti-solvent process had a significant impact on the morphology and optical properties of MAPbBr_3_. Finally, we analyzed the performance of the PSCs based on the relationship between the structural and electrical properties of the MAPbBr_3_ thin films and achieved a high power conversion efficiency (PCE) of 7.58% using the n-i-p normal planar structure.

## 2. Materials and Methods

### 2.1. Device Fabrication

All the chemicals were used as received, that is, without purification. To prepare the precursor solution, titanium diisopropoxide, 1-butanol, lead(II) bromide (PbBr_2_), N, N-dimethylformamide (DMF), and dimethyl sulfoxide (DMSO) were purchased from Sigma-Aldrich (Saint Louis, MO, USA). Methyl-ammonium bromide (MABr) was purchased from GreatCell Solar (Queanbeyan, Australia). For the fabrication of PSCs, patterned FTO-coated glass substrates (15 mm × 15 mm) were cleaned in detergent and rinsed with deionized water. Subsequently, the FTO glass substrates were sequentially ultrasonically cleaned with acetone, ethyl alcohol, and isopropyl alcohol, rinsed with deionized water, and dried in an N_2_ gas flow. The samples were then treated with an ultraviolet (UV) ozone lamp for 10 min to remove any organic residue. To prepare the TiO_2_ solution, 0.15 M of titanium diisopropoxide bis(acetylacetonate) (75 wt% in isopropanol) was mixed with 1-butanol. The TiO_2_ solution was spin-coated onto the cleaned FTO substrates at 700 rpm for 8 s, 1000 rpm for 10 s, and 2000 rpm for 40 s. The substrates were then dried on a hotplate at 125 °C for 5 min, heated to 450 °C for 60 min, and cooled to room temperature. The MAPbBr_3_ precursor solutions were prepared by dissolving MABr and PbBr_2_ in different molar ratios (0.5:1, 0.75:1, 1:1, and 1.25:1) in DMF and DMSO (4:1 *v*/*v*). The MAPbBr_3_ precursor solution was deposited using a one-step spin-coating method at 4000 rpm for 30 s. Next, 300 μL CB as anti-solvent was dripped onto the rotating substrate. After the deposition process, the substrate was heated at 100 °C for 10 min. The Spiro-OMeTAD (2,2′,7,7′-tetrakis[N,N-di(4-methoxyphenyl)amino]-9,9′-spirobifluorene) solution consisted of 73 mg of Spiro-OMeTAD, 26.6 mg of 4-tert-butyl pyridine, and 22.9 mg of a lithium bis(trifluoromethanesulfonyl)imide (Li-TFSI) solution (520 mg of Li-TFSI in 1 mL of acetonitrile) in 1 mL CB. The Spiro-OMeTAD layer was spin-coated as the HTL on the FTO/TiO_2_/MAPbBr_3_ substrate at 3000 rpm for 30 s. Finally, a 100-nm-thick Ag electrode was deposited by thermal evaporation on the top of the devices. All the devices were fabricated in an N_2_-filled glove box.

### 2.2. Device Characterization

The surface morphologies of the OIHP films were analyzed using field-emission scanning electron microscopy (FE-SEM). A high-resolution X-ray diffraction (HR-XRD) analysis system was used to determine the structural properties of the OIHP films. The optical properties of the OIHP films were measured using an ultraviolet-visible (UV-Vis) spectrophotometer, and their photoluminescence (PL) spectra were measured using a microscope equipped with a 405-nm laser. The sample structure used for the PL measurements was glass/MAPbBr_3_. The photocurrent density-voltage (*J-V*) characteristics of the solar cells were measured under air mass (AM) 1.5G 1-Sun illumination (100 mW/cm^2^), which was calibrated with a standard silicon reference solar cell and a photometer using a Keithley 2400 source meter.

## 3. Results and Discussion

### 3.1. Effects of MABr Content

**Figure 1:**
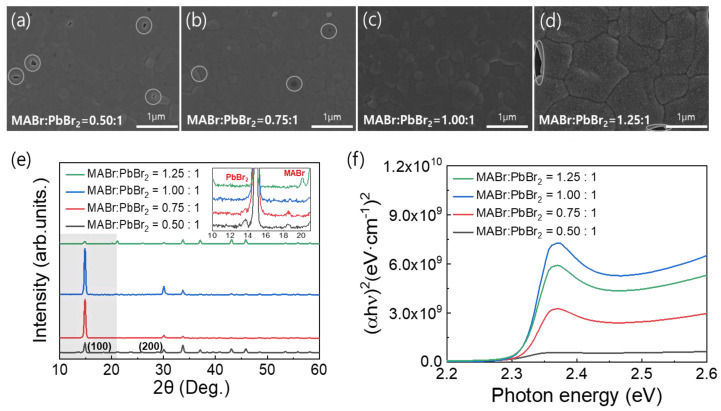
(**a**–d) Top-view SEM images, (**e**) XRD spectra, and (**f**) UV-Vis absorption spectra of MAPbBr_3_ films formed using precursor solutions with different MABr contents.

Figure 1a–d shows top-view SEM images of the MAPbBr_3_ thin films on the FTO substrates formed using the precursor solutions with the different MABr contents. These images clearly show distinct variations in the film morphologies. As shown in Figure 1a–d, an increase in the MABr content of the precursor solution increased the surface grain size of the coated MAPbBr_3_ thin film. However, pinholes were observed due to incomplete surface coverage except for the 1:1 molar ratio of MABr:PbBr_2_. As a result of the observed pinholes, we determined that the optimal MABr:PbBr_2_ molar ratio for producing the MAPbBr_3_ thin film was 1:1, as pinholes can create shunt paths in the PSCs. Dense and pinhole-free MAPbBr_3_ thin films are essential for improving the performance of PSCs. Figure 1e shows the XRD patterns of the MAPbBr_3_ OIHP films with the different MABr contents. For all the samples, strong peaks were observed at 14.90° and 30.05°, which corresponded to the (100) and (200) lattice planes, respectively, thus confirming the formation of a cubic perovskite structure [35]. However, in the case of the coatings formed using solutions containing an excessive amount of PbBr_2_ or MABr, the conversion of the OIHP film was incomplete. Therefore, the corresponding PbBr_2_ and MABr peaks are presented in the inset of Figure 1e [36,37,38]. The peak intensity of the film with a MABr:PbBr_2_ molar ratio of 1:1 was higher than other ratios (1.25:1, 0.75:1, and 0.5:1 molar ratio of MABr:PbBr_2_), which may be attributed to the complete crystallization of the film. Furthermore, the absence of any additional peaks in the XRD pattern of the film with 1:1 molar ratio of MABr:PbBr_2_ confirms its good crystallinity. The UV-Vis spectra in Figure 1f show the light absorption properties of the MAPbBr_3_ OIHP films with the different MABr contents. MAPbBr_3_ exhibits a characteristic absorption band at approximately 535 nm, which corresponds to a bandgap (E_g_) of 2.32 eV [12,39,40]. Changing the MABr molar ratio in perovskite precursor solution from 0.5 to 1 resulted in a slight increase in the absorbance, which then decreased when the molar ratio was changed further to 1.25. This change can be attributed to the crystallinity of the MAPbBr_3_ OIHP thin film [40].

**Figure 2:**
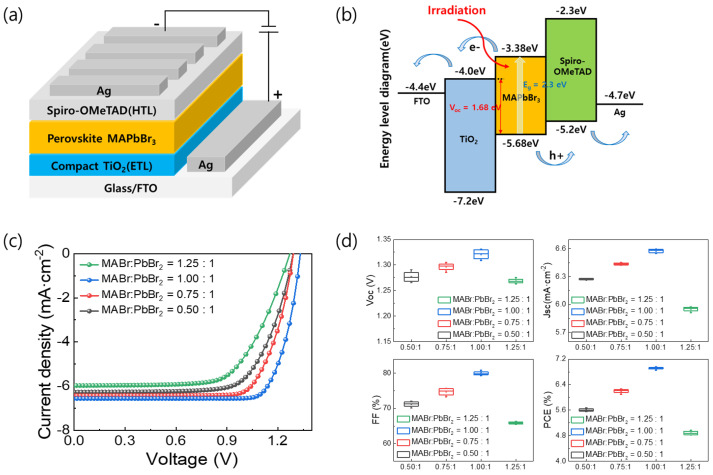
(**a**) Structure of MAPbBr_3_ PSCs. (**b**) Schematic of energy band diagrams of n-i-p device with FTO/TiO_2_/MAPbBr_3_/Spiro-OMeTAD/Ag structure. (**c**) *J-V* curves and (**d**) box charts of device parameters of MAPbBr_3_ PSCs formed using MABr:PbBr_2_ molar ratios of 1:0.5, 1:0.75, 1:1, and 1:1.25.

Figure 2a shows the schematic of the FTO/TiO_2_/MAPbBr_3_/Spiro-OMeTAD/Ag device. As noted previously, MAPbBr_3_ films with MABr:PbBr_2_ molar ratios of 0.5:1, 0.75:1, 1:1, and 1.25:1 in precursor solution were formed. Figure 2b shows the energy band diagrams of the PSCs based on the MAPbBr_3_ OIHP light absorber layer. As shown in Figure 2b, when light is incident on the MAPbBr_3_ light absorber layer, an electron-hole pair is created, and then the electron and hole can be well separated by the TiO_2_ and the Spiro-OMeTAD layer, respectively. Figure 2c shows the *J-V* characteristics of the PSCs depending on the MAPbBr_3_ layer coated with various MABr contents. The open-circuit voltage (*V_OC_*), short-circuit current density (*J_SC_*), fill factor (FF), and PCE of these devices are listed in Table 1. The device utilizing the MAPbBr_3_ film with a MABr:PbBr_2_ molar ratio of 1:1 exhibited the best photovoltaic performance due to its full surface coverage and good crystallinity. Specifically, it demonstrated *J_SC_*, *V_OC_*, FF, and PCE values of 6.55 mA·cm^−2^, 1.33 V, 79.87%, and 6.96%, respectively. As the MABr ratio was decreased, the device efficiency decreased slightly. In addition, the device efficiency decreased to 4.97% when the MABr:PbBr_2_ ratio was changed to 1.25:1. The *V_OC_*, FF, and PCE values are influenced by the quality of the perovskite light absorption film. Hence, the grain size and number of pinholes in the perovskite film have a determining effect on the device’s performance. The PCE, *J_SC_*, *V_OC_*, and FF were higher when the MABr:PbBr_2_ molar ratio was 1:1 due to the larger grain size of the MAPbBr_3_ film and its pinhole-free nature. Statistical values for the device parameters of the PSCs are shown in the box chart of Figure 2d.

### 3.2. Effects of Anti-Solvent Dripping Time

**Figure 3:**
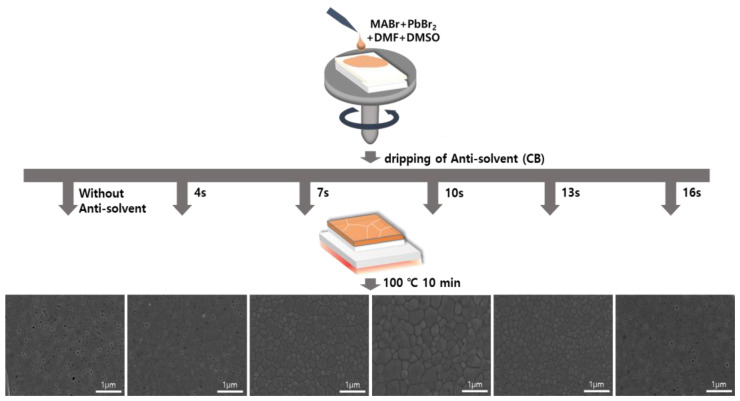
Schematic diagram of spin-coating process for forming MAPbBr_3_ thin films using different anti-solvent dripping times and SEM surface images of films formed for different anti-solvent dripping times.

A schematic for controlling the anti-solvent dripping time for an OIHP precursor solution fixed at an MABr:PbBr_2_ molar ratio of 1:1 is shown in Figure 3. The MAPbBr_3_ precursor solution was first dropped onto the FTO/TiO_2_ substrate and then subjected to spin-coating. The solvent of the MAPbBr_3_ solution evaporated during the spin-coating process, resulting in the formation of an OIHP layer. When the anti-solvent was not used, the unstable MAPbBr_3_ layer formed at the end of the spin-coating process and had low surface coverage. On the other hand, the drop of an anti-solvent during the spin-coating process of the MAPbBr_3_ solution resulted in the formation of a uniform, dense, and pinhole-free MAPbBr_3_ thin film due to rapid crystallization via improved nucleation and growth. This treatment facilitated the removal of the host solvent(s) and initiated the crystallization of the OIHP film. Therefore, high-quality MAPbBr_3_ OIHP films can be obtained by using an anti-solvent and optimizing its dripping time. To investigate the effects of anti-solvent dripping time on the quality of the OIHP films formed, five different dripping times (4, 7, 10, 13, and 16 s) were tested. Figure 3 shows top-view SEM images of the MAPbBr_3_ OIHP films formed using different dripping times. The MAPbBr_3_ films formed with dripping times of 7~13 s were uniform, dense, and flat. The film formed with a dripping time of 10 s had a larger grain size, homogeneous, and smoother surface. However, the films formed with dripping time of 4 and 16 s had low surface coverage, and numerous pinholes were observed. Therefore, the SEM results indicate that a dense and uniform MAPbBr_3_ film with a suitable grain size can be achieved using an appropriate anti-solvent dripping time.

**Figure 4:**
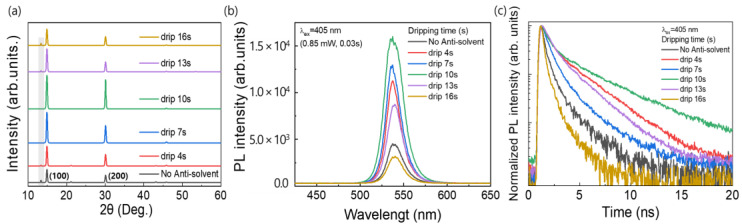
(**a**) XRD patterns, (**b**) PL, and (**c**) TRPL spectra of MAPbBr_3_ films formed using different anti-solvent dripping times.

Figure 4a shows the XRD patterns of the MAPbBr_3_ OIHP films formed using the different anti-solvent dripping times. For all the samples, strong peaks were observed at 14.90° and 30.05°, which corresponded to the (100) and (200) planes, respectively, and confirmed the formation of a cubic perovskite structure as mentioned above [35]. This means that a MAPbBr_3_ OIHP film was effectively formed irrespective of the anti-solvent dripping time. The anti-solvent not only promotes nucleation in the early stages of the film during spin-coating but also influences the initial crystal growth, which leads to a pinhole-free and homogeneous film morphology [41]. However, the peak intensity of the MAPbBr_3_ film formed without the anti-solvent process was decreased, indicating relatively poor crystallinity of the MAPbBr_3_ OIHP film. In addition, when the CB was dropped at 0, 4, 13, and 16 s, a peak related to PbBr_2_ was observed at 13.39°, indicating that partially unreacted PbBr_2_ remained in the perovskite film. Therefore, the optimal anti-solvent dripping time was determined to be 10 s. To investigate the luminescent properties of perovskite films prepared with various anti-solvent dripping times, steady-state PL and time-resolved photoluminescence (TRPL) were measured. Figure 4b shows the PL emission spectra of the MAPbBr_3_ films formed using the different anti-solvent dripping times. As shown in Figure 4b, the PL peak of all the MAPbBr_3_ samples occurred at 537.4 nm, as previously reported [42]. However, the PL emission intensity varied with the anti-solvent dripping time, and the film formed using a dripping time of 10 s showed the highest intensity. The strong PL emission could be attributed to the lower defect densities within the MAPbBr_3_ OIHP film [33]. Furthermore, Figure 4c shows the TRPL spectra of the MAPbBr_3_ films formed using the different anti-solvent dripping times. The TRPL curves are fitted with a bi-exponential function, where the fast decay component (*t*_1_) corresponds to the fast decay process, which is related to trap-assisted recombination, and the slow decay component (*t*_2_) corresponds to the slow decay process, which is related to radiative recombination [43,44]. The parameters of TRPL measurement for OIHP films made from various anti-solvent dripping times are summarized in Table 2. For OIHP made from anti-solvent dripping times of 10 s, the *t*_1_, *t*_2_, and average lifetime (*t*_ave_) are 0.59 ns, 3.80 ns, and 1.77 ns, respectively.

**Figure 5:**
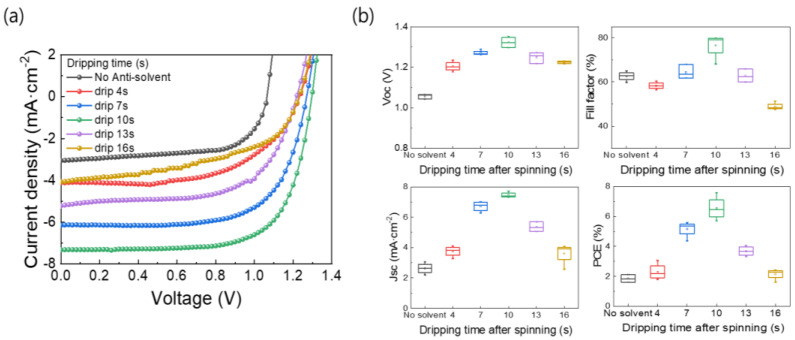
(**a**) *J-V* curves and (**b**) box charts of device parameters of MAPbBr_3_ PSCs based on films formed using different anti-solvent dripping times.

Figure 5a shows the *J-V* curves of the MAPbBr_3_ PSCs based on the films formed using the different anti-solvent dripping times. The device parameters are listed in Table 3, and Figure 5b shows the statistical data for the device parameters. As shown in Figure 5b, the overall device parameters were high for the MAPbBr_3_ PSC based on the film produced using an anti-solvent dripping time of 10 s. While the PSC based on the film produced without using an anti-solvent exhibited *J_SC_*, *V_OC_*, FF, and PCE values of 3.05 mA·cm^−2^, 1.06 V, 64.98%, and 2.11%, respectively, the device based on the film produced using an anti-solvent dripping time of 10 s showed values of 7.32 mA·cm^−2^, 1.30 V, 79.87%, and 7.58%, respectively. The device characteristics are consistent with the previous analysis results of the MAPbBr_3_ OIHP light absorber layer obtained through SEM, XRD, and PL measurements.

## 4. Conclusions

The effects of the MABr content (i.e., MABr:PbBr_2_ molar ratio) of the MAPbBr_3_ precursor solution and the anti-solvent dripping time during the spin-coating of the MAPbBr_3_ film on the performance of MAPbBr_3_-based PSCs were investigated in detail. Specifically, we investigated the effects of these parameters on the morphological, optical, and electrical properties of the MAPbBr_3_ OIHP films. The surface coverage and grain size of the MAPbBr_3_ films changed with the MABr:PbBr_2_ molar ratio in perovskite precursor solution and anti-solvent dripping time. The PSCs based on the MAPbBr_3_ film formed using the optimal MABr:PbBr_2_ molar ratio (1:1) and anti-solvent dripping time (10 s) showed the best performance, exhibiting *J_SC_*, *V_OC_*, FF, and PCE values of 7.32 mA·cm^−2^, 1.30 V, 79.87%, and 7.58%, respectively. Therefore, the molar ratio of MABr:PbBr_2_ and optimal anti-solvent treatment is important for preparing a high-quality and smooth form of MAPbBr_3_ thin film. In addition, we believe that our research makes a significant contribution to the wide-bandgap perovskite materials because it should aid the development of PSCs with improved performance.

## Figures and Tables

**Table 1:** Statistical device parameters of PSCs fabricated with MABr:PbBr_2_ ratios of 0.5:1, 0.75:1, 1:1, and 1.25:1.U+2.

MABr:PbBr_2_ Molar Ratio	PCE (%)	*V_OC_* (V)	*J_SC_* (mA/cm^2^)	FF (%)
0.50:1.00	5.67	1.29	6.26	70.11
0.75:1.00	6.26	1.29	6.42	75.61
1.00:1.00	6.96	1.33	6.55	79.87
1.25:1.00	4.97	1.27	5.97	65.45

**Table 2:** Time-resolved PL fitting parameters and average lifetimes of MAPbBr_3_ film fabricated with a different anti-solvent dripping time.

Anti-Solvent Time (s)	A_1_ (%)	*t*_1_ (ns)	A_2_ (%)	*t*_2_ (ns)	*t*_ave_ (ns)
No anti-solvent	25.04	0.29	0.37	1.59	0.39
drip 4	7.46	0.60	0.36	2.01	0.80
drip 7	4.35	0.72	0.52	2.99	1.47
drip 10	5.74	0.59	0.52	3.80	1.77
drip 13	3.48	0.86	0.45	2.68	1.38
drip 16	71.03	0.28	0.28	0.13	1.38

**Table 3:** Statistical device parameters of PSCs fabricated with a different anti-solvent dripping time.

Anti-Solvent Time (s)	PCE (%)	*V_OC_* (V)	*J_SC_* (mA/cm^2^)	FF (%)
No anti-solvent	2.11	1.06	3.05	64.98
drip 4	3.05	1.23	4.10	60.32
drip 7	5.41	1.27	6.27	67.92
drip 10	7.58	1.30	7.32	79.87
drip 13	4.05	1.22	5.33	62.37
drip 16	2.42	1.23	4.07	48.46

## Data Availability

The data that support the findings of this study are available from the corresponding author upon reasonable request.
